# Adolescents’ Responses to High-Intensity Versus Standard Physical Education on Body Fat, Blood Pressure, and VO_2_max: A Secondary Analysis Using TE-Based Responder Classification

**DOI:** 10.3390/healthcare14030410

**Published:** 2026-02-05

**Authors:** Jarosław Domaradzki, Eugenia Murawska-Ciałowicz, Katarzyna Kochan-Jacheć, Paweł Szkudlarek, Dawid Koźlenia, Marek Popowczak

**Affiliations:** Faculty of Physical Education and Sport, Wroclaw University of Health and Sport Sciences, 51-612 Wroclaw, Poland; jaroslaw.domaradzki@awf.wroc.pl (J.D.); eugenia.murawska-cialowicz@awf.wroc.pl (E.M.-C.); katarzyna.kochan-jachec@awf.wroc.pl (K.K.-J.); pawel.szkudlarek@op.pl (P.S.); marek.popowczak@awf.wroc.pl (M.P.)

**Keywords:** high-intensity interval training (HIIT), high-intensity plyometric training (HIPT), adolescents, cardiorespiratory fitness, preventive healthcare

## Abstract

**Highlights:**

**What are the main findings?**

- Both HIIT and HIPT delivered measurable improvements in body fat, blood pressure, and cardiorespiratory fitness among adolescents, with over half of the participants responding positively.
- Responsiveness was sex-dependent: males benefited more from HIIT, while females responded more strongly to HIPT, particularly in body fat reduction.

**What are the implications of the main findings?**

- Short, school-based high-intensity exercise protocols are effective tools for adolescent health promotion and cardiovascular risk prevention.
- Tailoring intervention type to sex may optimize health outcomes and improve the efficiency of preventive strategies in school settings.

**Abstract:**

**Background/Objectives**: A persistent challenge in adolescent health promotion is insufficient exercise intensity during physical education (PE) lessons, limiting their potential to reduce cardiometabolic risk. National curricula further restrict teacher flexibility in implementing effective preventive strategies. Brief, high-intensity exercise protocols may provide a scalable solution within school systems. Although their general effectiveness is established, less is known about the variability of individual health responses, particularly across multiple outcomes and in relation to sex and intervention type. This study aimed to (1) assess the prevalence of responders (Rs) and non-responders (NRs) by sex and intervention type, (2) examine sex-by-intervention interactions, and (3) evaluate the likelihood of combined positive health responses across body fat percentage (BFP), systolic and diastolic blood pressure (SBP and DBP), and cardiorespiratory fitness (maximal oxygen consumption [VO_2_max]). **Methods**: A total of 145 adolescents (aged 16 years; 48% males) from experimental school-based PE programs were analyzed. Two intervention modalities were implemented: high-intensity interval training (HIIT) and high-intensity plyometric training (HIPT). Rs were identified using typical error (TE) methodology. Statistical analyses included chi-squared tests (χ^2^), log-linear modeling, and odds ratios (ORs). **Results**: Chi-squared analyses indicated sex-by-intervention associations in the distribution of responder classifications for body fat percentage (BFP), systolic blood pressure (SBP), diastolic blood pressure (DBP), and VO_2_max (χ^2^ range = 8.26–10.10, *p* < 0.01). A simple association between intervention type and DBP was also observed (χ^2^ = 6.49, *p* = 0.011). However, logistic regression analyses yielded odds ratios with wide 95% confidence intervals crossing the null value for all outcomes, indicating limited precision and the absence of statistically robust interaction effects. Multinomial logistic regression examining combined responses (two or three concurrent improvements) revealed no statistically significant main or interaction effects (all *p* > 0.05). **Conclusions**: Brief high-intensity exercise protocols delivered within school-based physical education were associated with favorable changes in adiposity, blood pressure, and cardiorespiratory fitness in a substantial proportion of adolescents. However, sex- and intervention-specific differences in responder classification were not statistically significant and should be interpreted as exploratory. Further adequately powered studies are required to determine whether individual characteristics meaningfully moderate responsiveness to specific high-intensity exercise modalities.

## 1. Introduction

This manuscript presents a secondary analysis of data derived from the PEER-HEART randomized controlled trial (ClinicalTrials.gov ID: NCT06431230 date: 10 May 2024), which originally examined the effects of various school-based exercise programs on adolescent health. The current analysis focused exclusively on the two experimental arms—high-intensity interval training (HIIT) and high-intensity plyometric training (HIPT)—to examine inter-individual variability in training responsiveness and patterns of combined health outcomes. By isolating these intervention groups, the study allows for a more detailed evaluation of adaptation variability within active participants, although causal inference relative to non-intervention controls should be interpreted with caution.

Despite the well-documented benefits of regular physical activity, the World Health Organization reports that most adolescents fail to meet the daily physical activity recommendations—85% of girls and 78% of boys—contributing to early metabolic and cardiovascular dysfunctions [1,2]. These risk factors often develop during adolescence, a critical period for establishing lifelong health behaviors [3,4]. School-based physical education (PE) offers a unique, accessible, and sustainable platform for promoting physical activity and preventing cardiometabolic diseases on a population scale [5,6,7]. Due to its structured environment, universal coverage, and existing infrastructure, PE can integrate evidence-based training methods without requiring additional resources, making it a practical setting for implementing preventive interventions.

High-intensity training modalities, including HIIT and HIPT, have gained increasing attention for their efficiency and adaptability to school contexts [8,9,10,11,12,13,14,15,16,17,18,19,20,21,22,23]. HIIT consists of short bouts of vigorous effort interspersed with recovery, enhancing cardiovascular and metabolic health in a time-efficient manner [8,9,10,11,12,13,14]. HIPT, emphasizing eccentric–concentric muscle actions and neuromuscular engagement, can further improve strength, power, and body composition [14,15,16]. When applied within PE lessons, both approaches can complement the existing curriculum while promoting cardiovascular, muscular, and cognitive benefits [12,13,17,18,19,20].

Importantly, adolescents demonstrate considerable inter-individual variability in their physiological adaptations to training—known as individual variability in response to exercise training (IVRET) [21,22,23,24,25,26,27]. This phenomenon highlights that while many benefit substantially from structured exercise, others show limited or no measurable improvement. IVRET is influenced by a complex interplay of factors, including genetic predispositions, hormonal status, neuromuscular coordination, motivation, sleep, and nutrition [21,22,23,24]. Understanding these mechanisms and their role in shaping responders (Rs) and non-responders (NRs) is crucial for optimizing school-based interventions. The identification of Rs and NRs typically relies on the Typical Error (TE) approach, which allows for differentiation between meaningful biological adaptation and normal measurement variability [22,23,26].

Beyond mean group effects, biological sex has been proposed as a potential moderator of exercise responsiveness, particularly during adolescence, due to differences in hormonal milieu, muscle fiber composition, cardiovascular regulation, and metabolic responses [11,13,18,20,21,22,23,24]. While sex-related differences in average training adaptations have been examined primarily in adult populations, evidence remains limited regarding whether sex influences the likelihood of responding to distinct high-intensity exercise modalities in school-based settings, especially when responsiveness is defined using responder-based frameworks rather than mean group changes. Accordingly, the present study aimed to examine patterns of individual and combined health responses of normal-weight adolescents following two school-based high-intensity exercise programs (HIIT and HIPT). Specifically, we (1) assessed the prevalence of responders (Rs) and non-responders (NRs) by sex and intervention type, (2) explored sex-by-intervention associations in responder classification, and (3) evaluated the probability of combined positive responses across outcomes—body fat percentage (BFP), systolic and diastolic blood pressure (SBP and DBP), and maximal oxygen consumption (VO_2_max). We hypothesized that substantial inter-individual variability in responsiveness would be observed following both interventions and that sex and intervention modality might be associated with patterns of responder prevalence. 

## 2. Materials and Methods

### 2.1. Study Design

The present manuscript reports a planned secondary analysis focusing exclusively on the two intervention groups. All descriptive and inferential results reported in this article refer exclusively to participants in the HIIT and HIPT arms; control-class data are only referenced in relation to the parent RCT and are not re-analyzed here. The detailed methodology of the original randomized controlled trial has been previously reported [28]. In brief, the parent study used an 8-week, parallel-group, open-label design involving four intact school classes: two intervention groups (HIIT and HIPT) and two control groups. Randomization was implemented at the class level due to school organizational constraints; therefore, individual block randomization was not feasible, and sex distribution differed between classes. Nevertheless, all four classes followed identical teaching schedules and organizational structures.

The two control classes completed the standard school physical education curriculum, which consisted of team sports, endurance games, circuit-style calisthenics, and moderate-intensity running, without any structured high-intensity interval or power-based training. Thus, the key distinction between intervention and control classes was the presence (HIIT/HIPT) or absence (control) of a systematic high-intensity training stimulus.

The present manuscript reports a planned secondary analysis focusing exclusively on the two intervention groups, as only participants receiving the exercise stimulus could be classified as responders or non-responders. Data from baseline and post-intervention, assessments were used to quantify individual responsiveness. Because each school delivered a different intervention modality (HIIT vs. HIPT), school was treated as a fixed factor representing predefined intervention settings rather than randomly sampled clusters. Baseline comparisons showed no significant differences between the intervention groups, and therefore no additional multilevel adjustment was required. Control groups were not included in this secondary analysis because they did not receive an exercise stimulus and therefore could not be classified as responders or non-responders; the full intervention–control comparisons are reported in the primary trial publication [28]. Similarly, follow-up values were not included because the aim of this study was to quantify immediate responsiveness to the training stimulus, rather than the durability of adaptation. Long-term changes were reported in detail in the primary RCT article [28].

### 2.2. Clinical Trial Registration

This work and the subsequent results form part of a project funded by the state budget under the Polish Ministry of Science and Higher Education program “Science for Society II” (project no. NdS-II/SP/0521/2023/01). The study was registered as a clinical trial at ClinicalTrials.gov under the ID NCT06431230, with the acronym PEER-HEART (Physical Education dosE Response Health markErs Adolescents inteRval Training).

### 2.3. Ethics Committee

The Ethics Committee of the Wroclaw University of Health and Sport Sciences approved the study (approval number: ECUPE No. 33/2018, issued on 31 October 2018). The research complied with the ethical standards for medical research involving human participants, as stipulated by the World Medical Association in the Declaration of Helsinki.

### 2.4. Participants

The original randomized controlled trial included an a priori sample size calculation based on expected between-group differences in body fat and blood pressure between intervention and control classes. The full description is presented elsewhere [28]. The original G*Power (v.3.1) calculation was performed using a significance level of α = 0.05, statistical power of 0.80, and an expected medium effect size [29]. The present manuscript represents a secondary analysis restricted to the intervention arms and was therefore constrained by the available sample. No additional sample size calculation was performed specifically for the responder/non-responder analysis.

Therefore, the study sample included students from two schools, each taking part in one of the two forms of exercise, HIPT and HIIT. No adverse effects related to the interventions were observed, with the final analysis involving 145 adolescent students.

A total of 145 adolescents participated in the study, including 69 males and 76 females. Seventy-five students took part in the HIIT program (24 boys and 51 girls), and seventy students participated in the HIPT program (45 boys and 25 girls). The study was conducted in two public high schools located in the same city, characterized by comparable socioeconomic profiles, educational curricula, and access to sports facilities. Both schools followed the national PE framework, ensuring similar environmental and organizational conditions for implementing the intervention. Detailed information on socioeconomic status, race/ethnicity, and dietary habits was not collected because both participating schools served demographically comparable and socioeconomically homogeneous student populations within the same urban area. The absence of these variables is noted as a limitation of the study. Study participation was voluntary, with the option to withdraw at any time. The inclusion criteria for the HIIT group required participants to be first-year high school students regularly attending physical education (PE) classes. Eligible students had to be in good health, with no cardiovascular, respiratory, or musculoskeletal conditions that might limit their ability to safely perform high-intensity exercise. Additionally, participants could not be engaged in other structured high-intensity training programs outside the school curriculum. Participation was voluntary, with written informed consent obtained from all students and, in the case of minors, from their parents or legal guardians. Students and their parents or guardians were thoroughly briefed on the study’s aims and procedures. Written informed consent was obtained from the school principal, participants, and their parents or guardians prior to commencement.

### 2.5. Data Collection

#### 2.5.1. Procedures

Morphological measurements were conducted at three time points: before the intervention (Pre), immediately after the 8-week program (Post), and at the eight-week follow-up (FU). However, in the present secondary analysis, only the Pre and Post data were used, as the focus was on the immediate effects of the intervention rather than the long-term maintenance of change. All evaluations were carried out in the same sports halls between 8:00 a.m. and 1:00 p.m. under standardized conditions (temperature 20–22 °C, relative humidity 45–55%, stable lighting, and low noise levels). Participants wore T-shirts, shorts, and shoes during the general assessments, while anthropometric measurements were taken barefoot.

Body height (BH), body weight (BW), body composition, blood pressure (BP), and cardiorespiratory fitness were assessed in sequence. BP was measured on a separate day to ensure resting conditions and eliminate acute post-exercise effects that could bias cardiovascular readings. Body composition was evaluated using a calibrated bioelectrical impedance analyzer (Tanita MC-780, Tokyo, Japan), height with a stadiometer (Seca 213, Hamburg, Germany), and BP with an automated sphygmomanometer (Omron M6, Kyoto, Japan). VO_2_max was estimated via the multistage fitness test (MSFT), which demonstrates high validity and reliability in adolescents (ICC > 0.90). All devices were calibrated weekly according to the manufacturer’s recommendations.

Testing was conducted by experienced exercise physiologists and physical education specialists with over 5 years of field experience. Prior to data collection, all assessors underwent a training session to standardize testing procedures, achieving inter-rater reliability above 0.90 for repeated measures. Students, parents, and teachers were fully briefed on all testing guidelines before the project commenced.

#### 2.5.2. Body Morphology

Body height was measured to the nearest 0.1 cm using a GPM anthropometer (DKSH Ltd., Zürich, Switzerland) in a standing, barefoot position, following the International Society for the Advancement of Kinanthropometry (ISAK) guidelines [30]. Body weight and body fat percentage (BFP) were assessed using a Tanita Inner Scan V analyzer (BC-601, Tanita Co., Tokyo, Japan). All devices were calibrated before each testing session in accordance with the manufacturer’s recommendations. Participants received standardized pre-measurement instructions: they were asked to void their bladder, avoid excessive fluid intake, and maintain their usual breakfast routine. Measurements were performed in the evening, at least 3 h after the last meal, with participants barefoot and wearing minimal clothing. During assessment, heels were positioned on the rear electrodes of the scale, legs extended at the knees and hips, arms slightly abducted and flexed at the shoulders, elbows straight, and fingers in contact with the manual electrodes.

Body mass index (BMI) was calculated as body mass (kg) divided by height squared (m^2^).

#### 2.5.3. Blood Pressure

All blood pressure (BP) measurements were performed using a validated Omron BP710 Automatic Blood Pressure Monitor (Omron Healthcare, Inc., Hoffman Estates, IL, United States), compliant with the AAMI and ESH standards and demonstrating high validity and reliability for adolescent populations [31]. Appropriate cuff sizes were selected according to each participant’s upper arm circumference.

Participants were instructed to sit quietly with their back supported, legs uncrossed, and feet flat on the floor, avoiding conversation during measurement. After a 10-min seated rest period, three BP readings were taken at 10-min intervals in the same session, and the mean of the three values was used for analysis.

BP was assessed on a separate day to ensure resting conditions and to avoid potential acute effects of preceding physical activity or exercise testing. Measurements were conducted before and after the intervention period, as well as during the follow-up.

#### 2.5.4. Multistage Fitness Test

To assess maximal oxygen uptake (VO_2_max) and heart rate, the multistage fitness test (MSFT, also known as the 20 m shuttle run test) was administered using Polar Verity Sense sensors (Polar Electro, Kempele, Finland).

The MSFT protocol consisted of continuous running between two lines 20 m apart, synchronized with an audio signal. The initial running speed was 8.5 km·h^−1^, increasing by 0.5 km·h^−1^ every minute according to the standardized procedure [32]. The test was terminated when the participant failed twice consecutively to reach the line in time with the audio signal, indicating volitional exhaustion or inability to sustain the pace.

VO_2_max was estimated using the Ramsbottom formula [32], validated for adolescent populations:

VO_2_max = 3.46 × (L + SN/(L × 0.4325 + 7.0048)) + 12.2,

Testing was performed under standardized environmental conditions (temperature 20–22 °C, relative humidity 45–55%) and at the same time of day (8:00–11:00 a.m.) to control for circadian variation. Participants completed a 10-min standardized warm-up before the test and were instructed to give maximal effort, encouraged verbally throughout by the supervising instructor. Although respiratory exchange ratio (RER) and rate of perceived exertion (RPE) were not measured, the use of objective heart rate monitoring, standardized motivation cues, and termination criteria minimized subjectivity and ensured that the participants performed to volitional maximum [33].

### 2.6. Intervention

Both interventions were implemented twice weekly for eight weeks during regular physical education classes. Each session lasted 45 min and included a 10-min warm-up, a 20–25-min main part, and a 5-min cool-down. The HIIT program consisted of body-weight exercises such as squats, lunges, push-ups, and planks, performed in a Tabata format (20 s of maximal effort followed by 10 s of rest). The number of rounds progressed from four in weeks 1–2, to six in weeks 3–4, and eight in weeks 5–8. Exercise intensity was maintained at 75–85% of maximum heart rate (HRmax) using Polar Verity Sense monitors. The HIPT sessions followed the same Tabata structure and progression but included plyometric exercises such as jump squats, burpees, split jumps, and mountain climbers, alternating between upper- and lower-body movements to balance fatigue. All sessions were supervised by qualified instructors, and the total duration of each class remained consistent with the standard 45-min PE lesson. Intensity was monitored using Polar Verity Sense devices (Polar Electro, Kempele, Finland), with the target intensity set at 75% HRmax (determined from the MSFT) [32]. In other PE classes, students followed the school’s standard curriculum.

Each session included a 10-min standardized warm-up, followed by “all-out” HIIT or HIPT exercises performed in 4, 6, and 8 rounds across consecutive 2-week phases (Table 1). The HIIT program included squats, no-jump burpees, lunges, shoulder taps, lateral squats, push-ups, standing abdominal twists, and sit-ups. The HIPT program featured ankle hops, burpees, high knees, shoulder taps with claps, butt kicks, double-leg mountain climbers, squat jumps, and alternating-leg mountain climbers.

All sessions were supervised, with constant feedback and motivation. The control groups followed the standard PE curriculum focused on general fitness, team games, and individual disciplines, without structured high-intensity training.

Intervention was performed for two weeks using four rounds of 20 s of effort with 10 s of rest. In weeks 3–4, this increased to six rounds, and in weeks 5–8 to eight rounds.

The training intensity during the 8-week program was monitored using continuous heart rate (HR) recording and expressed as a percentage of maximal heart rate (%HRmax). Across the intervention period, both HIIT and HIPT sessions were performed at high physiological loads consistent with vigorous-to-near-maximal intensity exercise.

For the HIPT group, the weekly mean intensities ranged from 75.9% to 80.7% HRmax, with standard deviations of 6.6–9.5% and weekly minimum–maximum ranges typically between 49–97% HRmax depending on the week. The highest mean intensity was observed in week 1 (80.7%), while slightly lower relative intensities were recorded toward weeks 5–8 (75–78%).

For the HIIT group, mean weekly intensities ranged from 79.2% to 85.7% HRmax, with minimum–maximum values across the program typically falling between 41–97% HRmax. The highest mean intensity was recorded in week 1 (85.7% HRmax), and subsequent weeks consistently produced vigorous intensities between 79–82% HRmax.

All exercise sessions were conducted under the supervision of certified physical education teachers and exercise specialists. Participants were screened for any signs of illness or injury before each session, and only those cleared for participation were allowed to exercise. Proper technique and pacing were demonstrated and monitored throughout, with immediate first-aid support and medical assistance available on-site. No adverse events or injuries were reported during the intervention.

### 2.7. Classification of Responders and Non-Responders

Responders (Rs) and non-responders (NRs) were defined as individuals who did or did not experience beneficial changes following completion of the intervention [5,34]. To distinguish true change from measurement noise, the Typical Error (TE) was calculated separately for each outcome (fat mass, SBP, DBP, VO_2_max, and the BP composite) using the formula [6]:

TE = SDdiff/√2,

where SDdiff is the standard deviation of the Post–Pre difference scores. The TE thresholds are presented in the Supplementary Materials (Table S1). This approach was necessary because no repeated-baseline (Pre–Pre) measurements or independent test–retest dataset were available in the original trial. Consequently, the Post–Pre interval provided the only pair of repeated measurements suitable for estimating within-individual variability.

The TE method was chosen because it provides a distribution-free estimate of the within-individual variability expected from repeated measurements and is therefore well-suited to small and moderately sized samples, where traditional confidence-interval approaches may be unstable. By defining meaningful change as twice the TE, this method distinguishes genuine physiological responses from random measurement noise and has been widely recommended in previous exercise-science research.

Based on this TE value, individual responses were classified using the established ±2 × TE criterion:

- Responder (R): change > +2 × TE,
- Non-responder (NR): change within ±2 × TE,
- Reverse responder: change < −2 × TE.

This methodological framework follows that described previously [35,36], where also used TE and the ±2 × TE criterion to classify responders and non-responders. A change exceeding ±2 × TE is unlikely to arise from measurement error alone (approximate 95% confidence boundary), and therefore indicates a meaningful physiological response. Classification was outcome-specific and applied independently to each variable.

### 2.8. Statistics

The primary associations examined in this study were: (1) differences in responder status across sex and intervention modality, and (2) interaction effects between sex and intervention (SEX × INT) on responder outcomes (BFP, SBP, DBP, VO_2_max, BP composite). Categorical associations were evaluated using partial and marginal χ^2^ tests. To estimate effect sizes, logistic regression models were fitted for each responder outcome, including SEX, INT, and their interaction term. Logistic regression results are reported as odds ratios (ORs) with 95% confidence intervals and exact *p*-values.

Because some logistic regression effects yielded wide confidence intervals crossing the null value, interpretations of non-significant effects were made cautiously to avoid overstating the findings. All analyses were conducted using complete-case data, and statistical significance was interpreted in conjunction with effect size magnitude and precision.

Baseline descriptive characteristics of the full randomized controlled trial have been reported in detail in our previous publication [28]. The present work included only the frequencies of Rs and NRs in BFP, SBP, DBP, and VO_2_max. The main analyses concerned the prevalence of responder classifications and used χ^2^ tests, logistic regression, and log-linear models. These methods do not assume normality of the underlying data; therefore, minor deviations from normality in the continuous variables were not relevant to model selection. Continuous variables were summarized descriptively, but no distribution-dependent parametric testing was required for the primary research questions.

Analyses were conducted in the following steps:

Chi-squared tests were used to assess the independence of Rs and NRs for each variable. An omnibus χ^2^ test was first conducted to determine whether overall associations existed between categorical variables. When significant, the associations were further decomposed using partial and marginal χ^2^ tests. Partial χ^2^ tests assessed the unique contribution of each factor (sex, intervention modality) while controlling for the other, whereas marginal χ^2^ tests examined the bivariate associations without adjustment. This combination allowed us to distinguish general associations from factor-specific effects. All χ^2^ analyses met the required assumptions. Expected cell frequencies were checked for each contingency table, and no expected value was below 5; therefore, the χ^2^ test was considered appropriate and reliable for the present data.

The contingency coefficient (C) was calculated to assess associations, and odds ratios (ORs) estimated the likelihood of responses.

Sex moderation of intervention effects was analyzed using log-linear modeling of Rs/NRs frequencies [37,38,39].

In this study, “two responses” refer to beneficial changes in two of three physiological domains: body fat (BFP), blood pressure (BP), and cardiorespiratory fitness (VO_2_max). So, each combination of the two responses from all of the above listed was treated as “two responses” (BFP and BP, BFP and VO_2_max, BP and VO_2_max), while “three responses” refer to beneficial changes in all three physiological domains assessed: BFP and BP and VO_2_max. For combined responses, sex (SEX) and intervention type (INT) were used as predictors. Multinomial logistic regression tested associations between SEX/INT and combined responses.

All contingency tables were inspected to ensure that minimum cell frequencies satisfied the assumptions of χ^2^ testing and log-linear modeling (no expected cell counts < 5). As all outcomes met these criteria, no collapsing of categories or alternative procedures was required.

No additional covariates were included in the analyses because detailed information on potential confounders such as baseline fitness levels, socioeconomic status, or dietary habits was not available. The original class-level randomization helped minimize baseline imbalance, but unmeasured confounding cannot be fully ruled out.

No formal multiple-testing correction (e.g., Bonferroni adjustment) was applied because the analyses in this secondary study were exploratory and aimed at describing patterns of responsiveness rather than making confirmatory inferences. Accordingly, *p*-values should be interpreted with caution, and the emphasis is placed on effect size estimates (η^2^ for ANOVA-type comparisons; odds ratios with 95% confidence intervals for logistic models), which reflect the magnitude and precision of the observed effects.

Given the sample size and the secondary nature of the analysis, models were restricted to the primary factors of interest (sex, intervention modality, and their interaction) to minimize overfitting. Potential confounders such as BMI, adherence, and motivation were not included as covariates and are acknowledged as limitations.

The significance level was set at α = 0.05. All analyses were performed using Statistica V.13.0 (Tibco, Cracow, Poland).

## 3. Results

### 3.1. Participant Characteristics

A total of 145 adolescents (69 males, 76 females) completed the intervention and were included in the final analysis. Of these, 75 students participated in HIIT and 70 in HIPT. Baseline characteristics (body weight, height, BMI) were comparable between intervention groups within each sex (Table 1).

Baseline characteristics for the full randomized cohort have been reported in detail elsewhere, and the present manuscript includes only the subset of adolescents assigned to the intervention arms. Consistent with our previous article, the only statistically meaningful differences at baseline were those observed between sexes (sex: F = 54.571, *p* < 0.001, η^2^pG = 0.018, d = 0.19). In contrast, detailed post hoc comparisons indicated that none of the baseline variables differed significantly between the intervention subgroups (all *p* > 0.1).

Table 2 presents the Pre–Post changes (Δ) in body fat, blood pressure, and cardiorespiratory fitness for both intervention groups (HIIT and HIPT), shown separately for males and females. The Pre–Post changes displayed in Table 2 are provided descriptively; statistical comparisons of these deltas were already reported in the original RCT paper [28] and were not repeated in this secondary analysis.

### 3.2. Prevalence of the Responders and Non-Responders After Intervention Program

The numbers and percentages of Rs and NRs after the intervention program in each of the studied outcomes are presented in Table 1. These distributions were further analyzed using log-linear models to evaluate potential interactions between sex and intervention type.

Regardless of the type of intervention, the response to the exercise stimulus did not depend on sex. No significant association was found between sex and response status in any of the analyzed variables (Table 1). Intervention effects (irrespective of sex) differed by training modality, although not consistently across all outcomes. A statistically significant association was observed between intervention type and VO_2_max (*p* = 0.011), with HIPT showing a 2.3 times higher risk of non-response compared with HIIT. This value reflects the model-based odds ratio from the logistic regression analysis. The crude OR obtained directly from the raw frequencies (2.37) differed slightly from the model-derived estimate, which is expected given the adjustment inherent in the regression model. A near-significant trend was also found for DBP (*p* = 0.063). In this case, both interventions produced more responders than non-responders, but the proportions favored the HIPT group (Table 3).

The analyses above examined associations between each factor separately and the outcomes. However, these tests did not account for potential interactions between sex and intervention type. Therefore, further analyses were performed using the log-linear method to explore combined effects.

At the onset of log-linear analysis, model specifications were established with reference categories of female, HIIT, and response (1). An omnibus test was conducted to identify the model of effects to be tested. For BFP, the best-fitting model included the SEX × INT interaction and the main effect of responsiveness (RES) (χ^2^ = 4.11, *p* = 0.250). For SBP, the final model consisted of the SEXINT interaction and the main effect of RES (χ^2^ = 4.02, *p* = 0.259), and for DBP, the same structure was applied (χ^2^ = 4.11, *p* = 0.250). For VO_2_max, only the SEX*INT interaction was included (χ^2^ = 5.11, *p* = 0.751). All chi-squared tests were statistically non-significant, indicating satisfactory model fit to the observed data. Although χ^2^ tests suggested differences in responder distributions across sex and modality, logistic regression estimates showed modest effect sizes with 95% CIs crossing the null value (Table 4), indicating limited precision of these comparisons.

**Figure 1:**
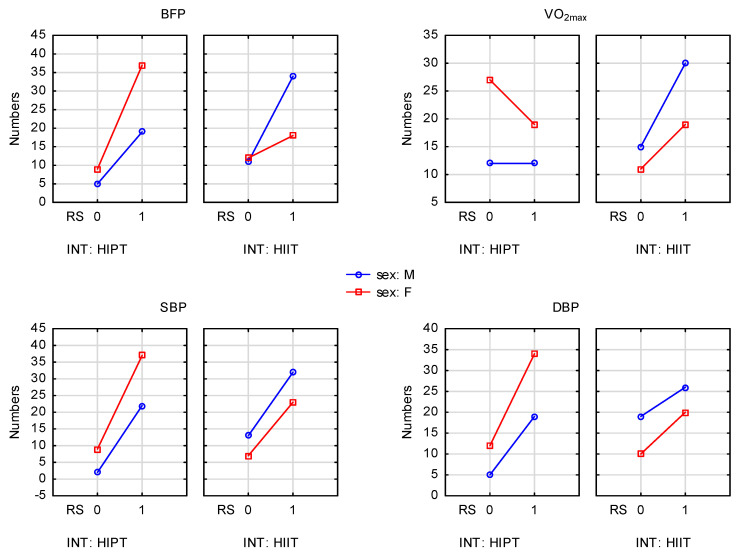
Interactions between the numbers of responders and non-responders across sex and intervention categories. BFP—body fat percentage; VO_2_max—maximal oxygen consumption; SBP—systolic blood pressure; DBP—diastolic blood pressure; RS—response: 1 = present, 0 = absent; INT—intervention type: HIPT = high-intensity plyometric training left panel; HIIT = high-intensity interval training—right panel. Axis X presents responder status (RS0 = non-responders; RS1 = responders); Axis Y presents proportion of participants (%). Blue color of the solid line is form males and red one is for females. The following group sizes are shown in the figure: HIPT males = 24 (16.6%), HIPT females = 46 (31.7%), HIIT males = 45 (31.0%), and HIIT females = 30 (20.7%). Percentages are relative to the total intervention sample (N = 145).

Figure 1 presents the interactions of responders and non-responders across sex and intervention categories.

Using partial and marginal chi-squared tests, the nature of the effects described above was examined in greater detail. The corresponding results are presented in Table 4. To improve clarity, Table 3 presents the main effects (SEX, INT, RES) and the interaction effect (SEX × INT) derived from the partial and marginal χ^2^ tests of the log-linear models. Partial χ^2^ values show the unique contribution of each factor after adjusting for the others, while marginal χ^2^ values reflect the bivariate associations.

All single main effects of sex and intervention type were non-significant, whereas the effect of response was significant for all outcomes except VO_2_max. The non-significant RES effect for VO_2_max (*p* = 0.2187) indicates that improvements in cardiorespiratory fitness were not systematically more or less common across sex or intervention type. In contrast to the other outcomes (BFP, SBP, DBP), the distribution of VO_2_max responders appeared more balanced and showed greater inter-individual variability not attributable to sex or modality. In contrast, the SEX × INT interaction terms from the log-linear (categorical) analyses were statistically significant for BFP, SBP, DBP, and VO_2_max (Table 4), indicating that responder distributions differed across sex-by-intervention subgroups in these categorical tests. 

However, logistic regression interaction estimates were accompanied by wide 95% confidence intervals crossing the null value (Table 5), and none of the regression interaction terms reached statistical significance. For interaction effects, odds ratios (ORs) were calculated using multiple logistic regression with the SEX*INT interaction term. The estimated odds ratios suggested directional tendencies in subgroup comparisons (e.g., HIPT vs. HIIT within sex); however, all corresponding 95% confidence intervals crossed the null value and the interaction terms were not statistically significant (Table 5). Although χ^2^ tests indicated statistically significant SEX × INT interactions in the categorical distributions of responders (Table 4), the corresponding logistic regression interaction coefficients were accompanied by 95% confidence intervals crossing the null value (Table 5). This difference reflects the fact that χ^2^ tests assess marginal associations between categories, whereas logistic regression estimates conditional interaction effects. As shown in Table 5, none of the logistic regression interaction terms reached statistical significance.

Table 4 shows the distribution of participants achieving 0, 1, 2, or 3 beneficial responses across the four intervention subgroups. One response was defined as a beneficial change in exactly one domain (BFP, BP, or VO_2_max); two responses were defined as concurrent improvements in any two domains (FAT–BP, FAT–VO_2_max, or BP–VO_2_max); and three responses were defined as beneficial changes in all three domains (FAT–BP–VO_2_max). This table is descriptive and intended to summarize the prevalence of combined outcomes prior to multinomial regression modeling. One response is noted as: FAT, BP, or VO_2_ max; two responses a: BFP –BP, FAT–VO_2_ max, or BP–VO_2_ max; and three responses as FAT–BP–VO_2_ max. Results are presented by sex and intervention type.

Among the males, 41.7% of those in the HIPT group and 33.3% in the HIIT group demonstrated full responses across all three outcomes. Additionally, 50% of HIPT males and 55.6% of HIIT males achieved two positive responses. In contrast, 19.6% of females in the HIPT group and 36.7% of those in the HIIT group achieved full responses, while 71.7% of HIPT females and 40.0% of HIIT females achieved two responses.

Table 6 descriptively summarizes combined responder patterns across subgroups. Given the non-significant multinomial model terms and wide confidence intervals, subgroup differences presented in Table 6 should be interpreted cautiously.

The final stage of the analysis assessed the likelihood of achieving two or three positive responses according to sex and intervention type. Participants with no response and those with a single response were combined into one reference category (“at most one response”). Multinomial logistic regression was applied, with two responses and three responses modeled against the reference. The results are presented in Table 7.

Multinomial logistic regression produced odds ratios for each response combination; however, none of the main effects or interaction terms reached statistical significance (all *p* > 0.05), despite the ORs indicating directional tendencies. The odds ratios in Table 5 represent the model-based estimates and may differ slightly from crude differences observed in Table 4; however, their associated *p*-values indicate that these effects were not statistically robust. For three responses versus at most one response, the OR of 0.97 indicated no meaningful interaction effect between sex and intervention type. Males were 34% more likely than females to be categorized as having three responses rather than at most one (OR = 1.34), while HIPT participants were 9% more likely than HIIT participants to achieve three responses (OR = 1.09).

For two responses versus at most one response, the interaction term was non-significant, suggesting no moderation effect of sex on intervention type. Males were 15% more likely than females to achieve two responses compared with at most one (OR = 1.15), and HIPT increased the odds of two responses by 28% relative to HIIT (OR = 1.28).

The HIPT group showed a lower proportion of VO_2_max responders in the combined response analysis (44.3%) compared with the HIIT group; however, this difference was not statistically significant and the wide confidence intervals suggest substantial uncertainty, meaning that these patterns should be interpreted cautiously. Although this descriptive difference suggests a weaker cardiorespiratory adaptation to HIPT, it did not reach statistical significance in the multinomial logistic regression model (all *p* > 0.05), and therefore should be interpreted with caution.

## 4. Discussion

The present study assessed the prevalence of responders (Rs) and non-responders (NRs) by sex and intervention type, examined sex-by-intervention interactions, and evaluated the likelihood of combined positive responses across body fat percentage (BFP), systolic and diastolic blood pressure (SBP and DBP), and cardiorespiratory fitness (VO_2_max). The results showed that the proportions of Rs and NRs for each outcome were similar between males and females, with no main effect of sex on responsiveness. Although sex-by-intervention associations were identified in the categorical χ^2^ analyses, logistic regression estimates were characterized by wide 95% confidence intervals crossing the null value, indicating limited precision of these effects. Accordingly, any apparent sex- or modality-related patterns should be interpreted as descriptive tendencies rather than statistically robust interaction effects. Multinomial logistic regression further indicated no statistically significant main or interaction effects for combined responder outcomes.

It is important to acknowledge that the original randomized controlled trial, from which the present data were derived, did not show between-group differences in the primary outcomes. Accordingly, the current study should not be interpreted as demonstrating differential effects of HIIT versus HIPT. Rather, this secondary analysis was designed to explore individual variability in responsiveness within the intervention arms. In line with this, none of the odds ratios exceeded the threshold of statistical significance, and any apparent patterns across sex or modality should be interpreted with caution. These findings highlight the considerable inter-individual variability in adolescents’ responses to high-intensity school-based exercise while underscoring the need for replication in adequately powered designs.

Both interventions elicited beneficial changes in more than 60% of participants, except for VO_2_max in the HIPT group, where the responder rate was 44.3%. The lower percentage of VO_2_max responders in the HIPT group may hypothetically be related to the neuromuscular rather than aerobic emphasis of plyometric training. However, because these differences were not statistically significant and the confidence intervals crossed the null value, this potential explanation should be regarded as speculative. Unlike HIIT, which directly stimulates maximal oxygen uptake through repeated bouts of high metabolic demand, HIPT relies on short, explosive actions with limited engagement of the aerobic system. Accordingly, any modality-related differences in VO_2_max responsiveness require confirmation in adequately powered prospective studies. Given that the regression-based interaction terms were not statistically significant and confidence intervals were wide, the following considerations are offered solely as potential hypotheses rather than explanatory conclusions. The observed sex-by-intervention interaction may be explained by several underlying mechanisms. Hormonal and neuromuscular differences between males and females likely modulate training adaptations. Higher testosterone concentrations in males promote muscle hypertrophy, hemoglobin synthesis, and anaerobic metabolism, facilitating performance gains in HIIT protocols characterized by repeated, high-intensity efforts [11,24,40]. In contrast, females typically exhibit a greater proportion of type I muscle fibers, higher fatigue resistance, and estrogen-mediated vascular and metabolic protection, which may enhance responsiveness to plyometric, bodyweight-based HIPT sessions [40,41,42]. Additionally, differences in motor coordination and movement efficiency could further contribute to inter-individual variability in adaptation patterns, rather than reflecting consistent sex-specific effects.

Both HIIT and HIPT protocols were associated with improvements in body composition and blood pressure, consistent with previous reports indicating that high-intensity school-based exercise can yield measurable health benefits in adolescents [11,43,44,45]. The current results align with previous evidence showing that regular HIIT reduces adiposity and blood pressure through mechanisms related to improved vascular function and autonomic regulation [43,44,46], while plyometric training enhances muscular efficiency and may indirectly support cardiovascular adaptations [42,47,48].

Because several of the estimated odds ratios were associated with wide confidence intervals, the precision of the responder estimates varied across outcomes. For this reason, the present study cannot provide a definitive sample size target for future research. Instead, future trials should base their sample size calculations on the effect-size ranges and confidence intervals reported here, ideally after replication in independent cohorts. This approach will allow for a more accurate estimation of the sample sizes required to detect sex- or modality-specific differences in responsiveness with adequate statistical power.

The beneficial effects of high-intensity exercise interventions observed in this study may plausibly involve several interrelated mechanisms. Physiologically, repeated exposure to high-intensity effort enhances endothelial function and vascular elasticity through increased nitric oxide bioavailability, leading to reduced peripheral resistance and blood pressure [42,43,44,45,46]. Metabolic adaptations include improved mitochondrial density, insulin sensitivity, and lipid oxidation, which contribute to reductions in body fat and overall cardiometabolic risk [43,44,45]. Both HIIT and HIPT stimulate the recruitment of fast-twitch muscle fibers and increase motor unit synchronization, improving power output and muscle efficiency [14,15,16,48]. Psychologically, engaging high-intensity activities may support motivation and adherence through perceived challenge and novelty. Collectively, these mechanisms are consistent with the direction of changes observed in the parent trial; however, they should be regarded as supportive context rather than definitive explanations of the present responder-based findings. This study has several limitations. As in our previous work [49], only post-pubertal adolescents were included, excluding prepubertal and peripubertal groups. Pubertal maturation can significantly influence metabolic and training responses, and future studies should account for developmental stage. Because this was a secondary analysis limited to the intervention groups, random allocation did not act as a balancing mechanism. Therefore, unmeasured factors such as BMI, adherence, motivation, or baseline fitness could have contributed to responsiveness. These factors could not be fully controlled and should be considered when interpreting sex-related differences. Another important limitation is that some potential confounding factors, such as habitual physical activity levels, socioeconomic background, and psychosocial variables, could not be analytically controlled due to the study design and logistical constraints. Therefore, their influence on the observed outcomes cannot be completely excluded and should be acknowledged when interpreting the results. This study did not include dietary assessment, which may have influenced cardiovascular and metabolic outcomes. Future research should incorporate dietary data collection and analysis to better control for the potential confounding effects of nutrition on training adaptations. Although the Typical Error (TE) method accounts for individual variability, it may not fully separate true biological adaptations from random fluctuations, regression to the mean, or placebo effects. Thus, some observed changes may reflect statistical rather than physiological responses. Because multiple statistical tests were conducted, an increased risk of type I error cannot be ruled out; however, as this was an exploratory secondary analysis, results were interpreted with caution and with primary emphasis on effect sizes and confidence intervals rather than on isolated *p*-values. As a secondary analysis, the study was limited by the absence of detailed information on potential confounders, such as baseline fitness, adiposity profiles beyond basic anthropometrics, socioeconomic status, or lifestyle factors. Although the class-level randomization in the parent trial reduced initial imbalance, residual confounding cannot be excluded, and the findings—particularly comparisons across sex or modality—should be interpreted with caution. Because multiple statistical tests were performed, the risk of type I error cannot be ruled out. As this secondary analysis was exploratory, no Bonferroni or other multiplicity corrections were applied. Therefore, conclusions—particularly those involving marginal associations—should be interpreted cautiously. The focus on effect size estimates (η^2^, odds ratios, and 95% confidence intervals) provides a more robust basis for interpretation than *p*-values alone. This secondary analysis did not investigate the baseline predictors of responder status (e.g., initial fitness, adiposity, or BMI). Subgroup sample sizes (sex × intervention) were insufficient for stable multivariable modeling, and attempting predictive analyses would risk model overfitting and unreliable estimates. Future studies with larger samples are needed to determine whether baseline characteristics can meaningfully predict responsiveness to school-based high-intensity training. Another limitation is the potential decrease in motivation associated with repeated high-intensity sessions. Although earlier studies reported good adherence [49], in the present trial, occasional signs of participant boredom were observed. Ensuring long-term engagement will therefore require careful monitoring of program attractiveness and variety when implementing HIIT and HIPT in school settings.

## 5. Conclusions

Brief high-intensity exercise programs conducted within school-based physical education were associated with favorable changes in body fat, blood pressure, and cardiorespiratory fitness in a substantial proportion of adolescents, with more than half of the participants classified as responders for at least one outcome. Although sex-by-intervention associations were observed in categorical analyses, regression-based models yielded wide confidence intervals and no statistically robust interaction effects. Therefore, any observed patterns of responsiveness should be interpreted as exploratory and descriptive rather than confirmatory. These findings highlight considerable inter-individual variability in adolescents’ responses to high-intensity exercise and underscore the need for adequately powered, prospectively designed studies to determine whether individual characteristics meaningfully moderate training responsiveness.

## Figures and Tables

**Table 1:** Descriptive statistics of the basic anthropometric measures pre- and post-intervention per sex and variant.

Males		HIIT	HIPT	Females		HIIT	HIPT
		mean ± sd (95% CI)	mean ± sd (95% CI)			mean ± sd (95% CI)	mean ± sd (95% CI)
Age [years]	Pre	15 ± 0.6 (14.8–15.3)	14.9 ± 0.5 (14.8–15.1)	Age [years]	Pre	15.1 ± 0.5 (14.9–15.2)	14.8 ± 0.4 (14.6–14.9)
Bh [cm]	Pre	175.4 ± 6.6 (172.6–178.2)	176 ± 6.1 (174.2–177.9)	Bh [cm]	Pre	165.6 ± 6 (163.9–167.4)	162.9 ± 5.4 (160.8–164.9)
	Post	175.7 ± 6.6 (172.9–178.4)	176.3 ± 6.1 (174.5–178.2)		Post	165.8 ± 5.9 (164–167.6)	163.2 ± 5.4 (161.2–165.2)
Bw [kg]	Pre	65.5 ± 9 (61.7–69.3)	65.2 ± 11.6 (61.7–68.7)	Bw [kg]	Pre	58.2 ± 10.9 (55–61.5)	54.2 ± 6.5 (51.8–56.7)
	Post	64.9 ± 7.7 (61.6–68.1)	64.5 ± 10.6 (61.4–67.7)		Post	58.4 ± 9.9 (55.4–61.3)	54 ± 5.9 (51.9–56.2)
BMI [kg/m^2^]	Pre	21.3 ± 2.8 (20.1–22.5)	21 ± 3.3 (20–22)	BMI [kg/m^2^]	Pre	21.2 ± 3.6 (20.1–22.3)	20.5 ± 2.4 (19.6–21.4)
	Post	21 ± 2.5 (20–22.1)	20.7 ± 3 (19.8–21.6)		Post	21.2 ± 3.2 (20.2–22.2)	20.3 ± 2.1 (19.5–21.1)

Footnote: HIIT—High Intensity Interval Training variant of intervention, HIPT—High Intensity Plyometric Training variant of intervention, Bh—body height, Bw—body weight, BMI—body mass index.

**Table 2:** Mean changes (Δ), standard deviations, and 95% confidence intervals for body fat percentage, blood pressure (SBP, DBP), and VO_2_max from Pre to Post in the HIIT and HIPT intervention groups. HIPT: males *n* = 24, females *n* = 46; HIIT: males *n* = 45, females *n* = 30.

Variable	Intervention	Males	Females
		Mean ± SD (95% CI)	Mean ± SD (95% CI)
ΔBFP [%]		−0.67 ± 1.55 (−1.32–−0.01)	−1.54 ±2.66 (−2.33–−0.75)
ΔSBP [mm/Hg]	HIPT	−5.37 ± 4.32 (−7.2–−3.55)	−3.07 ± 4.7 (−4.46–−1.67)
ΔDBP [mm/Hg]		−2.62 ± 6.25 (−5.27–0.02)	−2 ± 5.09 (−3.51–−0.49)
ΔVO_2max_ [ml/kg/min]		2.14 ± 3.91 (0.49–3.79)	0.67 ± 2.84 (−0.17–1.52)
ΔBFP [%]		−1.31 ± 1.66 (−1.81–−0.81)	−0.63 ± 2.37 (−1.52–0.25)
ΔSBP [mm/Hg]	HIIT	−2.73 ± 5.28 (−4.32–−1.15)	−4 ± 6.89 (−6.57–−1.43)
ΔDBP [mm/Hg]		−0.22 ± 5 (−1.72–1.28)	−1.37 ± 5.63 (−3.47–0.74)
ΔVO_2max_ [ml/kg/min]		3.81 ± 4.67 (2.41–5.21)	0.41 ± 3.25 (−0.8–1.63)

Footnote. Table 2 presents descriptive Pre–Post change values (Δ) for contextual reference only. Inferential statistical comparisons of these delta values were conducted and reported in detail in the primary trial publication [28] and are not duplicated here, as the present study focused on the prevalence of responders and non-responders. Abbreviations: BFP—body fat percentage; SBP—systolic blood pressure; DBP—diastolic blood pressure; VO_2_max—maximal oxygen consumption; HIIT—high-intensity interval training; HIPT—high-intensity power training.

**Table 3:** Numbers and frequencies of individuals who gained (responders [Rs]) and did not gain (non-responders [NRs]) effects in body fat, blood pressure, and cardiorespiratory fitness based on sex and intervention type. OR is presented together with 95% CI (in brackets).

		Outcome							
		BFP		SBP		DBP		VO_2_max	
SEX	M (*n*,%)	16 (23.2)	53 (76.8)	15 (21.7)	54 (78.3)	24 (34. 8)	45 (65.2)	27 (39.1)	42 (60.9)
	F (*n*,%)	21 (27.6)	55 (72.4)	16 (21.1)	60 (79.0)	22 (29.0)	54 (71.1)	38 (50.0)	38 (50.0)
statistic		*χ*^2^ = 0.38, *p* = 0.540 C = 0.05 OR = 0.79 (0.37–1.68)		*χ*^2^ = 0.01, *p* = 0.912 C = 0.01 OR = 1.04 (0.47–2.31)		*χ*^2^ = 0.57, *p* = 0.451 C = 0.06 OR = 1.31 (0.65–2.64)		*χ*^2^ = 1.72, *p* = 0.189 C = 0.11 OR = 0.64 (0.33–1.24)	
INT	HIPT (*n*,%)	14 (20.0)	56 (80.0)	11 (15.7)	59 (84.3)	17 (24.3)	53 (75.7)	39 (55.7)	31 (44.3)
	HIIT (*n*,%)	23 (30.7)	52 (69.3)	20 (26.7)	55 (73.3)	29 (38.7)	46 (61.3)	26 (34.7)	49 (65.3)
statistic		*χ*^2^ = 2.17, *p* = 0.108 C = 0.13 OR = 0.51 (0.26–1.21)		*χ*^2^ = 2.58, *p* = 0.141 C = 0,12 OR = 0.56 (0.23–1.16)		*χ*^2^ = 3.46, *p* = 0.063 C = 0.15 OR = 0.51 (0.24–1.04)		*χ*^2^ = 6.49, *p* = 0.011 C = 0.21 OR = 2.37 (1.21–4.63)	

Footnote: INT—intervention type; −—lack of positive changes; +—positive changes; HIIT—high-intensity interval training; HIPT—high-intensity plyometric training; M—male; F—female; BFP—percentage of body fat; SBP—systolic blood pressure; DBP—diastolic blood pressure; FI—fitness index; VO_2_max—maximal oxygen consumption; OR—odds ratio; ()—brackets: 95% CI, C—contingency coefficient. Participant numbers for each subgroup were: HIPT males = 24 (16.6%), HIPT females = 46 (31.7%), HIIT males = 45 (31.0%), and HIIT females = 30 (20.7%). Percentages are relative to the total intervention sample (N = 145). Abbreviations: BFP—body fat percentage; SBP—systolic blood pressure; DBP—diastolic blood pressure; VO_2_max—maximal oxygen consumption; HIIT—high-intensity interval training; HIPT—high-intensity power training.

**Table 4:** Partial (χ^2^ _part_) and marginal (χ^2^ _marg_) chi-squared tests examining associations between sex, intervention type, and responder classification for body fat percentage (BFP), systolic blood pressure (SBP), diastolic blood pressure (DBP), and VO_2_max. Main effects and selected interactions are shown.

		BFP				SBP		
Effect	χ^2^ _part_	*p*	χ^2^ _marg_	*p*	χ^2^ _part_	*p*	χ^2^ _marg_	*p*
SEX	0.3	0.5663			0.33	0.5663		
INT	0.2	0.6821			0.17	0.6821		
RES	35.2	0.0000			48.98	0.0000		
SEX × INT	10.1	0.0015	9.44	0.0021	9.52	0.0020	9.44	0.0021
		DBP				VO_2max_		
SEX	0.33	0.5663			0.33	0.5663		
INT	0.17	0.6821			0.17	0.6821		
RES	19.27	0.0000			1.51	0.2187		
SEX × INT	8.98	0.0027	9.443	0.0021	8.26	0.0041	9.44	0.0021

Footnote. Identical *p*-values for SEX and INT reflect identical χ^2^ statistics arising from the distribution of responder outcomes across categories, and are not typographical errors. SEX, INT, and RES represent main effects for sex, intervention type, and responder status, respectively. SEX × INT represents the two-way interaction. Partial χ^2^ values reflect adjusted effects; marginal χ^2^ values reflect unadjusted associations. Abbreviations: BFP—body fat percentage; SBP—systolic blood pressure; DBP—diastolic blood pressure; VO_2_max—maximal oxygen consumption; HIIT—high-intensity interval training; HIPT—high-intensity power training.

**Table 5:** Sex × intervention-type interactions for responder classification across physiological outcomes (BFP, SBP, DBP, VO_2_max), presented as odds ratios with 95% confidence intervals.

Outcome	Interaction Term (Sex × School)	β	SE (β)	OR	95% CI	*p*
BFP	Sex (M vs. F) × School (17 vs. 7)	0.802	0.806	2.23	0.46–10.82	0.320
SBP	Sex (M vs. F) × School (17 vs. 7)	−1.273	0.989	0.28	0.04–1.95	0.198
DBP	Sex (M vs. F) × School (17 vs. 7)	−0.673	0.779	0.51	0.11–2.35	0.387
VO_2_	Sex (M vs. F) × School (17 vs. 7)	−0.205	0.707	0.81	0.20–3.26	0.772
BP (SBP or DBP)	Sex (M vs. F) × School (17 vs. 7)	−1.517	1.302	0.22	0.02–2.82	0.244

Abbreviations: BFP—body fat percentage; SBP—systolic blood pressure; DBP—diastolic blood pressure; VO_2_max—maximal oxygen consumption; HIIT—high-intensity interval training; HIPT—high-intensity power training.

**Table 6:** Frequencies and percentages of participants achieving combined responder outcomes: no response, single response (body fat, blood pressure, or VO_2_max), two responses (FAT–BP, FAT–VO_2_max, or BP–VO_2_max), and three responses (FAT–BP–VO_2_max).

Sex	INT	NO Response	One Response			Two Responses			Three Responses
			FAT	BP	VO_2_max	FAT–BP	FAT–VO_2_max	BP–VO_2max_	FAT–BP–VO_2_max
M	HIPT	1	0	1	0	9	1	2	10
*n* = 24	%	4.2%	0.0%	4.2%	0.0%	37.5%	4.2%	8.3%	41.7%
M	HIIT	0	1	2	2	12	6	7	15
*n* = 45	%	0.0%	2.2%	4.4%	4.5%	26.7%	13.3%	15.6%	33.3%
K	HIPT	0	2	2	0	24	3	6	9
*n* = 46	%	0.0%	4.4%	4.4%	0.0%	52.2%	6.5%	13.0%	19.6%
K	HIIT	1	1	3	2	6	0	6	11
*n* = 30	%	3.3%	3.3%	10.0%	6.7%	20.0%	0.0%	20.0%	36.7%

Footnote: INT refers to the intervention modality and includes two categories: HIIT (high-intensity interval training) and HIPT (high-intensity power training). Rows represent the four intervention subgroups (HIIT males, HIIT females, HIPT males, HIPT females). Columns represent the number of outcomes achieved (0, 1, 2, or 3 responses). Values are frequencies (%). Abbreviations: BFP—body fat percentage; SBP—systolic blood pressure; DBP—diastolic blood pressure; VO_2_max—maximal oxygen consumption; HIIT—high-intensity interval training; HIPT—high-intensity power training.

**Table 7:** Multinomial logistic regression results for one and two responses compared to three responses by gender and intervention type.

			β	*p*		95%	
	Effect	Level			OR	Low	High
SEX	M	Three	0.29	0.2974	1.34	0.77	2.30
INT	7	Three	0.09	0.7551	1.09	0.63	1.88
SEX*INT	1	Three	−0.03	0.9027	0.97	0.56	1.67
SEX	M	Two	0.14	0.6069	1.15	0.68	1.93
INT	7	Two	0.24	0.3573	1.28	0.76	2.14
SEX*INT	1	Two	−0.40	0.1321	0.67	0.40	1.13

Abbreviations: BFP—body fat percentage; SBP—systolic blood pressure; DBP—diastolic blood pressure; VO_2_max—maximal oxygen consumption; HIIT—high-intensity interval training; HIPT—high-intensity power training.

## Data Availability

The data presented in this study are available on request from the author.

## Supplementary Materials

The following supporting information can be downloaded at: https://www.mdpi.com/article/10.3390/healthcare14030410/s1, Table S1: Tresholds (2*TE) for each outcome in males and females. Results for two modalities: HIIF and HIIT intervention.

## References

[1] World Health Organization (2018). Global Action Plan on Physical Activity 2018–2030: More Active People for a Healthier World.

[2] Dobbins M., Husson H., DeCorby K., LaRocca R.L. (2013). School-based physical activity programs for promoting physical activity and fitness in children and adolescents aged 6 to 18. Cochrane Database Syst. Rev..

[3] McGill H.C., McMahan C.A., Herderick E.E., Malcom G.T., Tracy R.E., Strong J.P. (2000). Origin of atherosclerosis in childhood and adolescence. Am. J. Clin. Nutr..

[4] Patton G.C., Coffey C., Carlin J.B., Sawyer S.M., Williams J., Olsson C.A., Wake M. (2011). Overweight and obesity between adolescence and young adulthood: A 10-year prospective cohort study. J. Adolesc. Health.

[5] A’Naja M.N., Reed R., Sansone J., Batrakoulis A., McAvoy C., Parrott M.W. (2024). 2024 ACSM worldwide fitness trends: Future directions of the health and fitness industry. ACSM’s Health Fit. J..

[6] Maturana F.M., Soares R.N., Murias J.M., Schellhorn P., Erz G., Burgstahler C., Widmann M., Munz B., Thiel A., Nieß A.M. (2021). Responders and non-responders to aerobic exercise training: Beyond VO2max. Physiol. Rep..

[7] Batrakoulis A., Jamurtas A.Z., Metsios G.S., Perivoliotis K., Liguori G., Feito Y., Riebe D., Thompson W.R., Angelopoulos T.J., Krustrup P. (2022). Comparative efficacy of five exercise types on cardiometabolic health in overweight and obese adults: A systematic review and network meta-analysis. Circ. Popul. Health Outcomes.

[8] Rodríguez-Fernández A., Lago Á., Ramirez-Campillo R., Sánchez M., Sánchez-Sánchez J. (2023). Cardiopulmonary-versus neuromuscular-based high-intensity interval training during a pre-season in youth female basketball players. Hum. Mov..

[9] Carson V., Rinaldi R.L., Torrance B., Maximova K., Ball G.D.C., Majumdar S.R., Plotnikoff R.C., Veugelers P., Boulé N.G., Wozny P. (2014). Vigorous physical activity and longitudinal associations with cardiometabolic risk factors in youth. Int. J. Obes..

[10] Gray S.R., Ferguson C., Birch K., Forrest L.J., Gill J.M. (2016). High-intensity interval training: Key data needed to bridge the gap from laboratory to public health policy. Br. J. Sports Med..

[11] Domaradzki J., Koźlenia D., Popowczak M. (2022). The mediation role of fatness in associations between cardiorespiratory fitness and blood pressure after high-intensity interval training in adolescents. Int. J. Environ. Res. Public Health.

[12] Peake J.M., Markworth J.F., Nosaka K., Raastad T., Wadley G.D., Coffey V.G. (2015). Modulating exercise-induced hormesis: Does less equal more?. J. Appl. Physiol..

[13] Eddolls W.T.B., McNarry M.A., Stratton G., Winn C.O.N., Mackintosh K.A. (2017). High-intensity interval training interventions in children and adolescents: A systematic review. Sports Med..

[14] Hammami M., Gaamouri N., Ramirez-Campillo R., Shephard R.J., Bragazzi N.L., Chelly M.S., Knechtle B., Gaied S. (2021). Effects of high-intensity interval training and plyometric exercise on the physical fitness of junior male handball players. Eur. Rev. Med. Pharmacol. Sci..

[15] Moran J., Sandercock G.R.H., Ramírez-Campillo R., Todd O., Collison J., Parry D.A. (2017). Maturation-related effect of low-dose plyometric training on performance in youth hockey players. Pediatr. Exerc. Sci..

[16] Racil G., Zouhal H., Elmontassar W., Abderrahmane A.B., De Sousa M.V., Chamari K., Amri M., Coquart J.B. (2016). Plyometric exercise combined with high-intensity interval training improves metabolic abnormalities in young obese females more so than interval training alone. Appl. Physiol. Nutr. Metab..

[17] Markovic G., Mikulic P. (2010). Neuro-musculoskeletal and performance adaptations to lower extremity plyometric training. Sports Med..

[18] Buchan D.S., Ollis S., Young J.D., Cooper S.M., Shield J.P., Baker J.S. (2013). High-intensity interval running enhances measures of physical fitness but not metabolic measures of cardiovascular disease risk in healthy adolescents. BMC Public Health.

[19] Cvetković N., Stojanović E., Stojiljković N., Nikolić D., Scanlan A.T., Milanović Z. (2018). Exercise training in overweight and obese children: Recreational football and high-intensity interval training provide similar benefits to physical fitness. Scand. J. Med. Sci. Sports.

[20] Delgado-Floody P., Latorre-Román P., Jerez-Mayorga D., Caamaño-Navarrete F., García-Pinillos F. (2019). Feasibility of incorporating high-intensity interval training into PE programs to improve body composition and cardiorespiratory capacity in overweight and obese children: A systematic review. J. Exerc. Sci. Fit..

[21] Martin-Smith R., Cox A., Buchan D.S., Baker J.S., Grace F., Sculthorpe N. (2020). High intensity interval training improves cardiorespiratory fitness in healthy, overweight and obese adolescents: A systematic review and meta-analysis of controlled studies. Int. J. Environ. Res. Public Health.

[22] Alvarez C., Ramirez-Campillo R., Ramírez-Velez R., Izquierdo M. (2017). Prevalence of non-responders in glucose control markers after 10-weeks of HIIT. Front. Physiol..

[23] Bonafiglia J.T., Rotundo M.P., Whittall J.P., Scribbans T.D., Graham R.B., Gurd B.J. (2016). Inter-individual variability in adaptive responses to endurance and sprint interval training: A randomized crossover study. PLoS ONE.

[24] Domaradzki J., Koźlenia D., Popowczak M. (2022). The Relative Importance of Age at Peak Height Velocity and Fat Mass Index in High-Intensity Interval Training Effect on Cardiorespiratory Fitness in Adolescents: A Randomized Controlled Trial. Children.

[25] Váczi M., Tollár J., Meszler B., Juhász I., Karsai I. (2013). Short-term high intensity plyometric training improves strength, power and agility in male soccer players. J. Hum. Kinet..

[26] Söyler M., Zileli R., Çingöz Y.E., Kılınçarslan G., Kayantaş İ., Altuğ T., Asan S., Şahin M., Gürkan A.C. (2024). Effect of high-intensity plyometric training on anaerobic performance in U17 elite A league: A pilot study. PeerJ.

[27] Davies G., Riemann B.L., Manske R. (2015). Current concepts of plyometric exercise. Int. J. Sports Phys. Ther..

[28] Domaradzki J., Popowczak M., Kochan-Jacheć K., Szkudlarek P., Murawska-Ciałowicz E., Koźlenia D. (2025). Effects of two forms of school-based HIIT on body fat, blood pressure, and cardiorespiratory fitness in adolescents: The PEER-HEART study. Front. Physiol..

[29] Faul F., Erdfelder E., Lang A.G., Buchner A. (2007). G*Power 3: A flexible statistical power analysis program for the social, behavioral, and biomedical sciences. Behav. Res. Methods.

[30] Marfell-Jones M., Olds T., Stewart A., Carter L. (2006). International Standards for Anthropometric Assessment.

[31] Nasir K., Ziffer J.A., Cainzos-Achirica M., Ali S.S., Feldman D.I., Arias L., Saxena A., Feldman T., Cury R., Budoff M.J. (2021). The Miami Heart Study (MiHeart): Rationale and design. Am. J. Prev. Cardiol..

[32] Ramsbottom R., Brewer J., Williams C. (1988). A progressive shuttle run test to estimate maximal oxygen uptake. Br. J. Sports Med..

[33] Duncombe S.L., Barker A.R., Price L., Walker J.L., Koep J.L., Woodforde J., Stylianou M. (2024). Was it a HIIT? A process evaluation of a school-based high-intensity interval training intervention. Int. J. Behav. Nutr. Phys. Act..

[34] Swinton P.A., Hemingway B.S., Saunders B., Gualano B., Dolan E. (2018). A statistical framework to interpret individual response to intervention. Front. Nutr..

[35] Bonafiglia J.T., Preobrazenski N., Gurd B.J. (2021). Systematic review examining approaches used to estimate interindividual differences in trainability. Front. Physiol..

[36] Domaradzki J., Koźlenia D., Popowczak M. (2023). Prevalence of responders and non-responders after 10 weeks of school-based HIIT. J. Clin. Med..

[37] Dobosz M. (2004). Computer Aided Statistical Analysis of Test Results.

[38] Daniel A., Freeman J. (1987). Applied Categorical Data Analysis.

[39] Burke P.J., Knoke D. (1986). Log-Linear Models.

[40] Martínez-Vizcaíno V., Sánchez-López M., Notario-Pacheco B., Salcedo-Aguilar F., Solera-Martínez M., Franquelo-Morales P., López-Martínez S., García-Prieto J.C., Arias-Palencia N., Torrijos-Niño C. (2014). Gender differences in effectiveness of a school-based PA intervention for reducing cardiometabolic risk. Int. J. Behav. Nutr. Phys. Act..

[41] Racil G., Coquart J.B., Elmontassar W., Haddad M., Goebel R., Chaouachi A., Amri M., Chamari K. (2016). Greater effects of high- vs moderate-intensity interval training on cardiometabolic variables in obese adolescent females. Biol. Sport.

[42] Ramírez-Campillo R., Abad-Colil F., Vera M., Andrade D.C., Caniuqueo A., Martínez-Salazar C., Nakamura F.Y., Arazi H., Cerda-Kohler H., Izquierdo M. (2016). Men and women exhibit similar acute hypotensive responses after plyometric training. J. Strength Cond. Res..

[43] Farah B.Q., Ritti-Dias R.M., Balagopal P.B., Hill J.O., Prado W.L. (2014). Does exercise intensity affect BP and HR in obese adolescents?. Pediatr. Obes..

[44] Da Silva M.R., Waclawovsky G., Perin L., Camboim I., Eibel B., Lehnen A.M. (2020). Effects of high-intensity interval training on endothelial function, lipid profile, body composition and physical fitness in adolescents. Physiol. Behav..

[45] Zeng Q., Dong S.Y., Sun X.N., Xie J., Cui Y. (2012). Percent body fat is a better predictor of cardiovascular risk factors than BMI. Braz. J. Med. Biol. Res..

[46] Costa E.C., Hay J.L., Kehler D.S., Boreskie K.F., Arora R.C., Umpierre D., Szwajcer A., Duhamel T.A. (2018). Effects of HIIT vs moderate continuous training on blood pressure in adults with hypertension. Sports Med..

[47] Uzor T.N., Uwa A.C., Ikwuka D.C. (2024). Effect of plyometric training on blood pressure of university athletes. Athena Phys. Educ. Sports J..

[48] Crozier J., Roig M., Eng J.J., MacKay-Lyons M., Fung J., Ploughman M., Bailey D.M., Sweet S.N., Giacomantonio N., Thiel A. (2018). High-intensity interval training after stroke: An opportunity to promote functional recovery and cardiovascular health. Neurorehabil. Neural Repair.

[49] Popowczak M., Rokita A., Domaradzki J. (2022). Effects of Tabata training on health-related fitness components among secondary school students. Kinesiology.

