# Supplementary material for: Adolescents’ Responses to High-Intensity Versus Standard Physical Education on Body Fat, Blood Pressure, and VO2max: A Secondary Analysis Using TE-Based Responder Classification
Source: Healthcare (Basel). 2026 Feb 5;14(3):410. doi: 10.3390/healthcare14030410 (PMC12897331; doi:10.3390/healthcare14030410)
Supplement: Supplementary file 1 [file healthcare-14-00410-s001.zip › healthcare-3919882-supplementary.pdf]

Table S1. Tresholds (2*TE) for each outcome in males and females. Results for two modalities: HIIF and HIIT intervention.

		Delta			
Modality	sex	BFP	SBP	DBP	FI
HIFT	M	2,193535	6,112176	8,844674	5,533847
	K	3,755961	6,649325	7,198765	4,020018
HIIT	M	2,351445	7,461416	7,070354	6,603873
	K	3,352474	9,741486	7,960968	4,601647
